# Evaluation of in vitro antioxidant activity of okra mucilage and its antidiabetic and antihyperlipidemic effect in alloxan‐induced diabetic mice

**DOI:** 10.1002/fsn3.2641

**Published:** 2021-10-28

**Authors:** A.F.M Irfan Uddin Zim, Jannatara Khatun, Mohammad Forhad Khan, Md. Altaf Hossain, Mohammad Mozibul Haque

**Affiliations:** ^1^ Department of Applied Food Science and Nutrition Faculty of Food Science and Technology Chattogram Veterinary and Animal Sciences University Khulshi, Chattogram Bangladesh; ^2^ Department of Animal Science & Nutrition Faculty of Veterinary Medicine Chattogram Veterinary and Animal Sciences University Khulshi, Chattogram Bangladesh; ^3^ Faculty of Food Science and Technology Chattogram Veterinary and Animal Sciences University Khulshi, Chattogram Bangladesh; ^4^ Department of Pharmacy International Islamic University Chittagong Chittagong Bangladesh; ^5^ Department of Pharmacy State University of Bangladesh Dhaka Bangladesh

**Keywords:** antidiabetic, antihyperlipidemic, antioxidant, mucilage, okra

## Abstract

Okra (*Abelmoschus esculentus*) is a traditionally important vegetable herb used to treat numerous illnesses, including diabetes mellitus, in many rural parts of Bangladesh and the South Asian subcontinent. However, the scientific evidence for the aforementioned properties has not been adequately validated. Hence, the aim of this study was to explore the antidiabetic, antilipidemic activity of okra mucilage powder, and to compare its effectiveness with the isolated peel–seed of okra after mucilage extraction in Swiss albino mice. After extraction, both mucilage and peel–seed were made into powder. In addition, crude protein, mineral contents, and in vitro antioxidant activity of mucilage and peel–seed powder were assessed. After acute toxicity test, methanolic extracts of both powders were administered to alloxan‐induced diabetic mice for 3 weeks. Blood glucose levels were assessed weekly. Finally, blood samples were collected on day 21 to estimate blood glucose level, total protein (TP), and lipid profile levels. Okra mucilage powder showed less amount of protein, calcium, magnesium, phosphorus, potassium, sodium, and iron compared with peel–seed powder. In terms of antioxidant activity, the IC50 value and total phenolic content were found higher in okra mucilage powder in contrast to peel–seed powder. However, total flavonoid content was higher in peel–seed powder than mucilage powder. Three‐week administration of mucilage and peel–seed suspensions at a dose of 150 mg/kg and 200 mg/kg significantly (*p* < .05) reversed the abnormal changes of bodyweights, water consumption, feed consumption, and fasting blood glucose levels of diabetic subjects. Cholesterol, triglycerides, low‐density lipoproteins, high‐density lipoproteins, and total protein were found to be significantly (*p* < .05) improved after mucilage and peel–seed treatment. Thus, Okra can be recommended as a potential source of antidiabetic drug candidate for the effective management of diabetes mellitus and its complications.

## Graphical abstract

The present research provides evidence that mucilage of *Abelmoschus esculentus* has antioxidant properties. The results from this study also revealed that the mucilage of *Abelmoschus esculentus* is rich in protein and minerals. Three weeks of administration of mucilage and peel–seed suspensions reduced the blood glucose concentration of alloxan‐induced diabetic mice. Cholesterol, triglycerides, low‐density lipoproteins, high‐density lipoproteins, and total protein were found to be significantly improved after mucilage and peel–seed treatment.

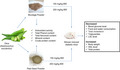

**Key Findings**

The present research provides evidence that mucilage of *Abelmoschus esculentus* has antioxidant property. The results from this study also revealed that the mucilage of *Abelmoschus esculentus* is rich in protein and minerals. Three weeks of administration of mucilage and peel–seed suspensions reduced the blood glucose concentration of alloxan‐induced diabetic mice. Cholesterol, triglycerides, low‐density lipoproteins, high‐density lipoproteins, and total protein were found to be significantly improved after mucilage and peel–seed treatment.

## 1 INTRODUCTION

Diabetes mellitus, a long‐term metabolic disease, is characterized by elevated glucose levels in the blood due to insulin secretion, insulin action, or both (Inzucchi et al., 2012). Insulin, which is synthesized by β‐cells of the pancreas, assists in maintaining glucose concentration in the blood by transporting it to the body cells and thus helps in producing energy (Ahmad, 2014). Hence, insulin resistance, dysfunction, or destruction of beta cell leads to aberrant glucose metabolism in the body causing diabetes mellitus (Kahn et al., 2014). People of low‐ and middle‐income countries are the most sufferers (Jalili & Niroomand, 2016). Rapid and ongoing socioeconomic transitions are also influencing the prevalence of diabetes in developing countries (Guariguata et al., 2014). Though an absolute cure has not been found yet, proper management can keep a diabetic patient healthy. General management of diabetes is defined by eating a healthy, nutritious diet, avoiding sugar‐rich food, maintaining a balanced weight, exercising in a regular manner, taking medications regularly and, if needed, taking insulin therapy. However, treatment with antidiabetic drugs is costly and that is the reason people in developing or low‐income countries always have an intention to heal the consequences or symptoms of diabetes in a more natural way. Therefore, current research focuses primarily on medicinal plants for the treatment of chronic diseases such as diabetes, with a focus on cost and side effect minimization, as well as also to make available this medicinal plant as ready form in the market.

Okra (*Abelmoschus esculentus*) is one of the traditional medicinal plant products that can be used in the management of diabetes. Given its robust nature, distinct seed, dietary fiber, and protein balance of both lysine and tryptophan amino acids, Okra has been called “a perfect villager's vegetable” (Holser & Bost, 2004). Okra has been found to be effective in the management of diabetes, digestive issues, colon health, the body's cholesterol level, and heart health (Gemede et al., 2015). Different parts of the okra plant have also revealed the presence of the total phenolics, total flavonoids, and antioxidant properties (Liao et al., 2012). Previous experiments have also demonstrated the in vivo and in vitro anti‐hyperglycemic as well as antihyperlipidemic activities of different parts of okra (Dubey & Mishra, 2018). Almost all parts of okra contain high amounts of mucilage (Ahiakpa et al., 2014; Sengkhamparn et al., 2009). Okra mucilage is generally an acidic polysaccharide and composed of galacturonic acid, galactose, rhamnose, arabinose, and glucose (Woolfe et al., 1977). Several experimental studies have suggested that it has potential as a food, non‐food product, and medicine (Gemede et al., 2015); (Kumar et al., 2010). In vitro studies have shown that okra polysaccharide has great potential in the management of hyperglycemia, oxidative stress, obesity, and weight gain because it inhibits glucosidase, amylase, lipase, and intestinal glucose absorption and has anti‐oxidative and muscle glucose uptake activity (Ozougwu et al., 2013). However, the experimental results of this study strongly suggested further in vivo antidiabetic activities of okra mucilage.

Therefore, the present study was conducted to determine the scientific basis of traditional uses and to evaluate hypoglycemic and hypolipidemic potential of raw okra mucilage in alloxan‐induced diabetic mice. Moreover, antioxidant potentiality and the effectiveness of okra mucilage and peel–seed of okra were investigated.

## 2 MATERIALS AND METHODS

### 2.1 Plant material/sample collection

Fresh fruits of Okra (*A. esculentus*) were procured from a local fruit and vegetable store in Chawkbazar market, Chattogram, Bangladesh. After that, the okra fruits were washed and stored in a refrigerator at 4°C until they were used.

### 2.2 Extraction of mucilage

The okra mucilage was extracted using the traditional method. Briefly, the okra was properly washed and soaked in distilled water for 8–9 h. Thereafter, it was heated in a water bath with continuous agitation for 30 min at 60°C to favor the thorough release of the mucilage into the water. The concentrated viscous solution was then filtrated through a muslin cloth, and the remaining okra fruits were isolated for further use. The filtrated viscous solution was cooled to room temperature. The filtered mucilage was spread on a non‐sticky paper over a tray. After that, the mucilage solution was dried to a constant weight in a cabinet dryer at 45°C for approximately 24 h. Later, the dried mucilage was ground into a fine powder with a mortar and pestle. The powdered mucilage (PM) was then passed through #80 sieve size and packed in airtight containers for further use and analysis.

### 2.3 Peel–Seed powder preparation

After the extraction of the mucilage, the separated fruits containing seeds were taken in a tray and washed with distilled water. Later, they were dried to constant weight at 45°C in a cabinet dryer. Then, the crispy fruits and seeds were ground into a fine powder using a mixer grinder. The powdered mixture (PPS) was then sieved through a # 80 mm sieve size before being stored in an airtight container for further study and analysis.

### 2.4 Methanolic extraction of PM and PPS

Methanolic extraction of powdered mucilage and powdered peel–seed mixture was done as described by (Yan et al., 2013). Dried plant materials (100 mg) were weighed into a conical flask. About 100 ml of 95% aqueous methanol was added. The suspension was stirred slightly in a water bath at below 40°C and then left at room temperature for 24 h. The extract was centrifuged for 10 min at 3000 rpm and then filtered through Whatman No. 4 paper. The supernatants were collected for use in an experiment.

### 2.5 Antioxidant activity

2,2‐diphenyl‐1‐picrylhydrazyl (DPPH) free radical scavenging activity of the test samples was determined using the method as described by (Akter et al., 2021; Gemede et al., 2018).

#### 2.5.1 Preparation of DPPH solution (100 µm)

At first, 4 mg of DPPH was dissolved in 100 ml of methanol (95%) in a dark condition.

#### 2.5.2 Preparation of standard ascorbic acid solution

To prepare stock solution of 1 mg/ml, about 10 mg of ascorbic acid was dissolved in 10 ml of distilled water. Then, serial dilution was performed in order to prepare different concentrated solutions (2, 4, 8, 16, and 32 µg/ml).

#### 2.5.3 Preparation of sample solution

Serial dilution was performed in order to prepare different concentrated solutions (2, 4, 8, 16, and 32 µg/ml).

#### 2.5.4 Procedure

About 4 ml of DPPH solution was added to 1 ml of sample extracts for standards at different concentration. The mixture was shaken vigorously and allowed to stand at room temperature in the dark for 30 min. Then, the absorbance of the solution was measured at 517 nm using a UV‐Vis spectrophotometer (Shimadzu UV‐2600, Japan) against blank. Control sample was prepared containing the same volume without any extract and reference ascorbic acid. Methanol was used as blank, and IC_50_ was calculated from % inhibition. Triplicate analysis was done for each sample. Scavenging of the DPPH free radical was measured using the following equation:

%inhibition={(A0‐A1)/(A0)}×100.

A0=absorbanceofDPPHalone.

A1sample=absorbanceofDPPHalongwithdifferentconcentrationsofextracts.

### 2.6 Determination of total phenol content

#### 2.6.1 Preparation of standard gallic acid solution

To prepare stock solution of 1 mg/ml, about 10 mg of gallic acid was dissolved into 10 ml of distilled water. Then, serial dilution was performed in order to prepare different concentrated solutions (2, 4, 8, 16, and 32 µg/ml).

#### 2.6.2 Procedure

The total phenol content of okra extracts was evaluated by the Folin–ciocalteu method as described by (Wojdyło et al., 2007). About 1 ml of sample extracts or standard at different concentrations was mixed with 2 ml of Folin–ciocalteu reagent (10 times diluted) and incubated at room temperature for 3 min. After that, 10 ml of 20% sodium carbonate was added to the mixture and left for incubation at room temperature for an hour. The absorbance of the mixture was measured at 765 nm with a Shimadzu UV–VIS‐2600 spectrophotometer against a blank solution. The blank solution contained all the reagent mixture without extract or standard sample. Gallic acid standard curve was used to quantify total phenolic contents, and the results were expressed as mg of gallic acid equivalent (GAE) per gram of dried weight. All determinations were performed in triplicate (*n* = 3).

### 2.7 Total flavonoid content determination

Flavonoid content in samples was measured by aluminum chloride colorimetric method as described by (Shah & Hossain, 2014).

#### 2.7.1 Preparation of standard quercetin solution

About 10 mg of quercetin was dissolved into 10 ml of distilled water. So, the concentration of the solution was 1 mg/ml. This is called stock solution. Then, serial dilution was performed in order to prepare different concentrated solutions (6, 12, 24, 48, and 96 µg/ml).

#### 2.7.2 Procedure

About 1 ml of sample or standard at different concentration solution was taken in a test tube. After that, 0.2 ml of 10% aluminum chloride, 0.2 ml of 1 M potassium acetate, and 8.6 ml of distilled water were added to each tube. The reaction mixture was then incubated at room temperature for 30 min to complete the reaction. The absorbance of the mixture was measured at 420 nm. Quercetin was used to make the calibration curve. The calculation of total flavonoids content in the extracts was carried out in triplicate, and the results were averaged. The final result was expressed as mg of quercetin equivalent (QE) per gram of dried weight. All determinations were performed in triplicate (*n* = 3).

### 2.8 Mineral analysis

Mineral contents were determined by using biochemical analyzer (Humalyzer 3000, Germany). Commercially available biochemical kit (Randox®, England) was used for biochemical assay. For sample preparation, 5 g of powdered sample was taken into a conical flask. After that, 7.5 ml HNO_3_ and 2.5 ml HClO_4_ were added into the conical flask. Then, it was heated over an induction cooker at 200 W until complete digestion. Then, it was cooled. Finally, deionized water was added up to 100 ml. The results were expressed as mg/100 g after conversion from mg/dl.

### 2.9 Crude protein determination

The crude protein was determined by Kjeldahl method (AOAC, 2016). About 0.3 g sample was weighted into digestion tube. A mixture containing 72 g potassium sulfate and 8 g copper sulfate was prepared. About 4 g of this mixture was added to the digestion tube. Then, 5 ml of concentrated H_2_SO_4_ was added into the digestion tube. Digestion was carried out at 320°C for 30 min. Sample was cooled down before addition of 25 ml of distilled water and 25 ml of 40% NaOH. About 10 ml 4% boric acid with three drops of green bromocresol indicator was prepared as receiving solution in conical flask. Cooled tube and receiving solution were placed into the distillation unit. After that, 25 ml 40% NaOH has filled automatically into the tube and the distillation process was conducted for 4 min. The receiving solution turned to green color after the distillation process. The receiving solution was titrated with 0.2 N HCL until it turned to gray color. Triplicate analysis was done for each sample. The percentage of crude protein was calculated by using the following formula.

Percentageofnitrogen=((T‐B)×N×14.007×100)/(Weightofsampleinmg).

T=Volumeoftitrationofsample.

B=VolumeoftitrationforBlank.

N=NormalityofHCL(0.2)

#### 2.9.1 Experimental animals and diet

About 60 healthy Swiss albino mice of both sexes, weighed between 23 and 27 g and 5 weeks old, were purchased from the animal house of the Department of Pharmacy, Jahangirnagar University, Bangladesh. The mice were raised in the Department of Animal Science and Nutrition's animal house at CVASU. Laboratory conditions with appropriate temperature, humidity, and a 12‐h light: 12‐h dark cycle were maintained. The mice were placed in standard ventilated cages, and free access to food and water was ensured. Mice were acclimatized for 7 days before the commencement of the study. The mice were fed a pellet diet for the duration of the study.

#### 2.9.2 Acute toxicity study

Acute oral toxicity test of okra powdered mucilage and powdered peel–seed mixture was carried out as per Organization for Economic Cooperation and Development Guidelines (OECD) guidelines (Guidance, 2001). Mice were divided into five groups, each group consisting of six animals. The PM and PPS were diluted with distilled water and administered orally at 100, 200, 400, 800, and 1000 mg/kg body weight. The animals were observed for 24 h for behavioral or any adverse change.

#### 2.9.3 Induction of diabetes

Overnight, fasted mice were selected for the diabetic group and intraperitoneally administered with alloxan monohydrate (150 mg/kg body weight) dissolved in ice‐cold saline (0.9% NaCl). To prevent alloxan‐induced hypoglycemia, the animals received a 5% glucose solution for the next 24 h. After 4 days of the injection of alloxan, the blood glucose level of the mice was recorded by using a glucometer. Experimental animals showed stabilized diabetes after the fourth day of the administration of alloxan. Mice having blood glucose levels higher than 7 mmol/L were considered as diabetic mice and were included in the present experiment. Animals which did not reveal signs of diabetes after the fourth day were withdrawn from the study (Tao Bu et al., 2012).

#### 2.9.4 Experimental design

All mice were divided into seven dietary groups with eight animals per group. All treatments were given using oral gavage for 3 weeks. Groups were included as follows: Group 1: Normal control (NC) treated with distilled water; Group 2: Diabetic control (DC) (alloxan‐induced diabetic mice) received only distilled water; Group 3: Diabetic mice treated with standard drug (SD) glibenclamide at a dose of 5 mg/kg body weight; Group 4: Diabetic mice treated with powdered peel–seed (PPS) at a dose of 150 mg/kg body weight; Group 5: Diabetic mice treated with powdered peel–seed (PPS) at a dose of 200 mg/kg body weight; Group 6: Diabetic mice treated with powdered mucilage (PM) at a dose of 150 mg/kg body weight; and Group 7: Diabetic mice treated with powdered mucilage (PM) at a dose of 200 mg/kg body weight. All mice were supplied normal pellet diet and ad libitum water. Suspension of powdered mucilage (PM), powdered peel–seed mixture (PPS), and glibenclamide were prepared with distilled water just before the oral administration. The bodyweight and fasting blood glucose levels of the mice were recorded every week during the experiment period. Feed consumption was recorded during the study period.

#### 2.9.5 Oral glucose tolerance test

Oral glucose tolerance test was conducted on overnight fasted control and treated mice after 3 weeks of administration. After measuring the fasting blood glucose level, glucose solution (2 g/kg body weight) was given to the animals by oral gavage. Blood was withdrawn again from the tail vein at 30, 60, 90, and 120 min after glucose administration by using glucometer (Gluco Dr, Korea; Tao Bu et al., 2012). Calculation of the area under the curve (AUC) was measured according to the method (Dong et al., 2014) using following formula:

$$\mathrm{Area}\;\mathrm{under}\;\mathrm{the}\;\mathrm{curve}=\left(\mathrm{Basal}\;\mathrm{glycemia}\;+\;\mathrm{glycemia}\;\mathrm{at}\;0.5\;\mathrm{hour}\right)\;\times 0.25+\left(\mathrm{glycemia}\;\mathrm{at}\;0.5\;\mathrm{hour}\;+\;\mathrm{glycemia}\;\mathrm{at}\;1\;\mathrm{hour}\right)\times 0.25+\left(\mathrm{glycemia}\;\mathrm{at}\;1\;h+\;\mathrm{glycemia}\;\mathrm{at}\;2\;\mathrm{hour}\right)\;\times 0.5.$$

#### 2.9.6 Collection of blood samples

At the end of the experiment, blood samples were collected by cardiac puncture from overnight fasting anesthetized (by diethyl ether) animals. Serum was separated from blood after 40–60 min of centrifugation at 3500 rpm for 10 min. The obtained serum samples were stored at −30°C until analysis.

#### 2.9.7 Biochemical tests

Total protein (TP), total cholesterol (TC), high‐density lipoprotein (HDL), and triglyceride (TG) levels were measured by Humalyzer 3000 using commercial kit from Randox laboratories limited (United Kingdom). The low‐density lipoprotein (LDL) levels were calculated according to the formula Friedewald et al. (1972) as follows:

LDL=Totalcholesterol‐(HDL+Triglyceride/5).

#### 2.9.8 Statistical analysis

All statistical analysis was done using statistical package for social sciences (SPSS) version 25. One‐way analysis of variance was used to evaluate the data. Data are presented as the mean ± Standard Error (SE). Differences in means were compared using the Tukey test. P values < .05 were considered significant.

## 3 RESULTS

### 3.1 In vitro antioxidant activity of okra mucilage and peel–seed

#### 3.1.1 Determination of DPPH scavenging activity

Results for the DPPH free radical scavenging activity of methanolic extracts of PM and PPS are shown in Table 1. Both PM and PPS showed a dose‐dependent radical scavenging effect in DPPH assay. The half inhibition concentration (IC_50_) value of ascorbic acid was 9.22 µg/ml. In contrast, the IC50 value for free radicals achieved by the PM and PPS is 73.83 and 67.09 µg/ml, respectively (Table 1). So, in comparison with ascorbic acid, it is clear that the peel–seed mixture possesses more anti‐radical activity than the mucilage.

**TABLE 1:** DPPH radical scavenging activity of mucilage and peel–seed

Serial No.	Concentration (µg/ml)	% Inhibition of Ascorbic acid	% Inhibition of mucilage	% Inhibition of peel–seed
1	2	28.78 ± 0.29^a^	10.39 ± 0.08^b^	5.58 ± 0.11^c^
2	4	37.63 ± 0.1^a^	15.01 ± 0.05^b^	13.09 ± 0.02^c^
3	8	54.67 ± 0.26^a^	22.23 ± 0.1^b^	20.59 ± 0.08^c^
4	16	69.59 ± 0.34^a^	24.83 ± 0.02^b^	23.68 ± 0.16^c^
5	32	90.57 ± 0.12^a^	27.23 ± 0.16^b^	26.66 ± 0.03^c^
IC_50_ (µg/ml)		9.22 ± 0.08^a^	73.83 ± 0.76^b^	67.09 ± 0.29^c^

Note

All data are expressed as mean ± SE. Means ± SE within the row bearing different superscripts (a, b, and c) is significantly different (*p* < .05).

#### 3.1.2 Total phenol content

Phenol content was measured by using gallic acid calibration curve. Total phenol content of the methanolic extract of PM and PPS was found at 68.84 ± 0.3 mg Gallic acid equivalent per gram and 65.98 ± 0.3 mg Gallic acid equivalent per gram (Table 2). A significant difference (*p* < .05) was found between them.

**TABLE 2:** Total phenol content, total flavonoid content, total protein content, and mineral content of mucilage and peel–seed

Sample^*^	Total Phenol Content (mg GAE/g)	Total Flavonoid Content (mg QE/g)	Total protein Content (g/100 g)	Sodium (mg/100 g)	Potassium (mg/100 g)	Calcium (mg/100 g)	Magnesium (mg/100 g)	Phosphorus (mg/100 g)	Iron (mg/100 g)
PM	68.84 ± 0.3^a^	7.90 ± 0.1^a^	8.54 ± 0.96^a^	5.72 ± 0.02^a^	112 ± 1.4^a^	120 ± 5.7^a^	196 ± 1.4^a^	50 ± 1.4^a^	1.03 ± 0.01^a^
PPS	65.98 ± 0.3^b^	9.50 ± 1.1^a^	11.28 ± 1.27^b^	5.17 ± 0.04^b^	422 ± 4.2^b^	344 ± 8.5^b^	324 ± 4.24^b^	306 ± 4.24^b^	1.15 ± 0.07^a^

Note

^*^PM, powdered mucilage and PPS, powdered peel–seed. The values are expressed as Mean ± SE. Means ± SE within the column bearing different superscripts (a and b) is significantly different (*p* < .05).

#### 3.1.3 Total flavonoid content

Flavonoid content was determined by using quercetin calibration curve. Total flavonoid content of the methanolic extract of peel–seed (PPS) mixture was 9.50 ± 1.1 mg Quercetin equivalent/g, and for mucilage (PM), the value was 7.90 ± 0.1 mg Quercetin equivalent/g (Table 2). No significant difference (*p* < .05) was observed between them.

### 3.2 Protein and mineral contents

Powdered peel–seed mixture had significantly (*p* < .05) higher amount of potassium, calcium, magnesium, and phosphorus compared with powdered mucilage (Table 2). Iron content was not differed between in PPS and PM. However, sodium content was found significantly higher in PM compared with PPS. In terms of protein, the crude protein contents of the peel–seed (PPS) were significantly higher (11.28 ± 1.27 g/100 g) than protein content in okra mucilage (PM) which was 8.54 ± 0.96 g/100 g (Table 2).

### 3.3 Toxicity study

The oral administration of mucilage powder and peel–seed mixture was found to be safe at a dose level of up to 1000 mg/kg of body weight in mice. Neither any toxicological effect nor mortality was observed. Finally, the dose of 150 and 200 mg/kg body weight was selected.

### 3.4 Food and water consumption of alloxan‐induced diabetic mice

**FIGURE 1:**
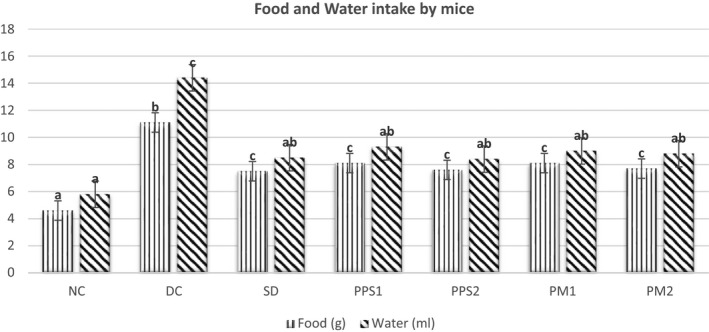
Average food and water intake of different mice group Legends: NC, Normal control; DC, Diabetic control; SD, Standard drug; PPS1, Peel–Seed (150 mg/kg); PPS2, Peel–Seed (200 mg/kg); PM1, Mucilage (150 mg/kg); PM2, Mucilage (200 mg/kg). Different Superscripts (a, b, c) within the food and water column denotes significant difference (*p* < .05) between different mice groups

Food intake and water consumption of alloxan‐induced diabetic mice are shown in Figure 1. The feed intake of DC was significantly higher (11.1 g/day) compared with NC mice which was around 4.6 g/day. However, the food intake was significantly lower (*p* < .001) in SD, PPS1, PPS2, PM1, and PM2 as compared to the diabetic group. In terms of water consumption, the normal control group drank only 5.8 ± 0.5 ml/day of water, which was statistically significant (*p* < .001) in contrast to the diabetic control groups (14.4 ± 0.7 ml/day). All other alloxan‐induced groups SD (8.5 ml/day), PPS1 (9.3 ml/day), PPS2 (8.4 ml/day), PM1 (9 ml/day), and PM2 (8.8 ml/day) consumed considerably lower amount of water when compared to the non‐treated diabetic control groups.

### 3.5 Effects of mucilage and peel–seed on body weight of mice

The effect of PPS and PM on the body weight of mice is shown in Table 3. The initial average body weight of all mice in various groups was about 25 g. The weight of normal control mice continued to increase evenly and the diabetic control group lost weight consistently to the end of the experiment. On week 1, no significant variation was noted between the DC and other treatment groups. However, at the end of the experiment, after third week, all of the mice receiving treatment had a significant (*p* < .05) increase in body weight when compared to the diabetic control.

**TABLE 3:** Effects of PPS and PM on body weight of mice

Sample^*^	Body weight in gram			
	Week 0	Week 1	Week 2	Week 3
NC	25 ± 0.5^a^	26.8 ± 0.5^b^	29.1 ± 0.4^a^	31.4 ± 0.6^a^
DC	25.6 ± 0.4^a^	24.7 ± 0.6^a^	22.9 ± 0.6^b^	21.8 ± 0.6^b^
SD	25.5 ± 0.2^a^	25.1±0.0.4^ab^	26.1 ± 0.4^c^	27.4 ± 0.9^c^
PPS1	25.4 ± 0.4^a^	25 ± 0.4^ab^	25.3 ± 0.4^c^	26.5 ± 0.5^c^
PPS2	25.1 ± 0.7^a^	24.9 ± 0.5^ab^	26.4 ± 0.4^c^	27.5 ± 0.6^c^
PM1	25.2 ± 0.4^a^	25.1 ± 0.4^ab^	25.6 ± 0.3^c^	27.1 ± 0.5^c^
PM2	25.3 ± 0.4^a^	25.1 ± 0.3^ab^	26.8 ± 0.6^c^	27.9 ± 0.6^c^

Note

^*^NC, Normal control; DC, Diabetic control; SD, Standard drug; PPS1, Peel–Seed (150 mg/kg); PPS2, Peel–Seed (200 mg/kg); PM1, Mucilage (150 mg/kg); and PM2, Mucilage (200 mg/kg). All data are expressed as mean ± SE. Means ± SE within the column bearing different superscripts (a, b, and c) is significantly different (*p* < .05).

### 3.6 Effect of mucilage and peel–seed on fasting blood glucose level

The fasting blood glucose level of mice each week is represented in Table 4. Distinct boost in blood glucose levels was observed in all samples induced by alloxan monohydrate. The fasting blood glucose level was around 4 mmol/L in all groups at initial stage. The glucose concentration in the blood increased from 12.3 ± 0.6 to 13.1 ± 0.8 mmol/L in different groups after alloxan induction. Furthermore, the diabetic control group's hyperglycemic effect increased significantly (*p* < .001) each week when compared to the normal control group. The blood glucose level was documented at 15.1 ± 0.5 mmol/L in DC group at the end of the experiment. However, the groups treated with a standard drug (glibenclamide), powdered mucilage (PM) suspension, and peel–seed mixture (PPS) suspension showed significant (*p* < .001) decrease in glucose levels over the 3‐week period when compared to DC group. Glibenclamide (5 mg/kg) treatment demonstrated the lowest blood glucose concentration in mice at 5.8 ± 0.3 mmol/L. Similarly, PPS1, PPS2, PM1, and PM2, after 3‐week treatment, resulted in 53.7%, 58.3%, 52.9%, and 55.6% reductions respectively in fasting blood glucose levels compared with non‐treated diabetic mice.

**TABLE 4:** Effect of PPS and PM on fasting blood glucose level in alloxan‐induced diabetic mice

Treatments^*^	Blood glucose (mmol/L)				
	Initial	Week 0	Week 1	Week 2	Week 3
NC	3.9 ± 0.1	4.2 ± 0.1^a^	4.3 ± 0.1^a^	4.4 ± 0.1^a^	4.5 ± 0.1^a^
DC	4.1 ± 0.1	12.3 ± 0.6^b^	14.1 ± 0.4^b^	14.6 ± 0.6^b^	15.1 ± 0.5^b^
SD	3.9 ± 0.1	12.9 ± 0.6^b^	8.2 ± 0.4^c^	6.9 ± 0.6^c^	5.8 ± 0.3^ac^
PPS1	4.2 ± 0.1	12.6 ± 0.7^b^	11.1 ± 0.3^d^	8.2 ± 0.2^c^	7 ± 0.1^c^
PPS2	3.9 ± 0.1	12.6 ± 0.3^b^	10.7 ± 0.3^d^	7.5 ± 0.7^c^	6.3 ± 0.5^c^
PM1	4.1 ± 0.2	12.3 ± 0.8^b^	10.9 ± 0.4^d^	8.3 ± 0.3^c^	7.1 ± 0.4^c^
PM2	4.2 ± 0.1	13.1 ± 0.8^b^	10.9 ± 0.4^d^	7.6 ± 0.5^c^	6.7 ± 0.4^c^

Note

^*^NC, Normal control; DC, Diabetic control; SD, Standard drug; PPS1, Peel–Seed (150 mg/kg); PPS2, Peel–Seed (200 mg/kg); PM1, Mucilage (150 mg/kg); PM2, Mucilage (200 mg/kg). All data are expressed as mean ± SE. Means ± SE within the column bearing different superscripts (a, b, and c) is significantly different (*p* < .05).

### 3.7 Oral Glucose Tolerance Test

The Oral Glucose Tolerance Test (OGTT) study supported the trends in the fasting glucose level test. The OGTT in mice demonstrated that blood glucose concentration in all animal groups reached the highest levels after 30 min of glucose administration (2 g/kg BW) and was found to have decreased steadily with time (Table 5). From Table 5, it is revealed that after 21 days of administration, glucose tolerance of the PPS and PM‐treated group showed the similar significant (*p* < .05) improvement as the standard drug glibenclamide when compared to the DC group (7.7 ± 0.2 to 8.9 ± 0.2 mmol/L versus 18.2 ± 0.5 mmol/L). The calculation of the AUC (area under the curve) also indicated a significant (*p* < .05) decrease of all treatment groups in contrast to the DC group. However, the AUC of all treatment groups was still considerably higher than that of the normal group.

**TABLE 5:** Effects of PPS and PM on oral glucose tolerance test

Treatments^*^	Oral Glucose Tolerance (mmol/L)				
	0 min	30 min	60 min	120 min	AUC
NC	4.5 ± 0.1^a^	12.2 ± 0.4^a^	7.6 ± 0.4^a^	4.6 ± 0.3^a^	15.2 ± 0.4^a^
DC	15.1 ± 0.5^c^	23.1 ± 0.5^c^	20.3 ± 0.7^c^	18.2 ± 0.5^c^	39.7 ± 0.6^d^
SD	5.8 ± 0.3^ab^	17.8 ± 0.4^b^	13.3 ± 0.4^b^	7.7 ± 0.2^b^	24.2 ± 0.4^b^
PPS1	7 ± 0.1^b^	18.9 ± 0.3^b^	15 ± 0.5^b^	8.6 ± 0.4^b^	26.8 ± 0.4^c^
PPS2	6.3 ± 0.5^b^	18.6 ± 0.5^b^	14.4 ± 0.6^b^	7.9 ± 0.3^b^	25.6 ± 0.6^bc^
PM1	7.1 ± 0.4^b^	19.1 ± 0.2^b^	14.6 ± 0.4^b^	8.9 ± 0.2^b^	26.7 ± 0.4^c^
PM2	6.7 ± 0.4^b^	18.8 ± 0.5^b^	14.3 ± 0.5^b^	8.3 ± 0.3^b^	25.9 ± 0.6^bc^

Note

^*^NC, Normal control; DC, Diabetic control; SD, Standard drug; PPS1, Peel–Seed (150 mg/kg); PPS2, Peel–Seed (200 mg/kg); PM1, Mucilage (150 mg/kg); and PM2, Mucilage (200 mg/kg). All data are expressed as mean ± SE. Means ± SE within the column bearing different superscripts (a, b, c, and d) is significantly different (*p* < .05).

### 3.8 Effect of mucilage and peel–seed on various biochemical parameters in mice

In terms of lipid profile, experimental mice with diabetes revealed significant increases in triglyceride, total cholesterol, low‐density lipoprotein (LDL) as well as significant reduction in high‐density lipoprotein (HDL) concentrations as compared to normal animals, as depicted in (Table 6). In diabetic mice, low‐density lipoprotein (LDL), total cholesterol, and triglycerides were significantly decreased as well as high‐density lipoprotein (LDL) levels increased significantly when compared to normal control group at all the experimental doses (Table 6). Mucilage as well as the peel–seed mixture significantly (*p* < .001) increased the HDL level and decreased the cholesterol, triglycerides, and LDL levels in alloxan‐induced diabetic mice compared with the diabetic control group (Table 6). It is also obvious that, at the same doses, PPS exerts a superior hypolipidemic effect than that of PM. Total protein in blood was significantly (*p* < .001) decreased in diabetic control mice than normal control mice. However, the total protein level was significantly (*p* < .001) increased after the administration of PM and PPS at the dose 150–200 mg/kg compared with diabetic control mice (Table 6).

**TABLE 6:** Effect of PM and PPS on various biochemical parameters in mice

Treatments^*^	Cholesterol (mg/dl)	TG (mg/dl)	HDL (mg/dl)	LDL (mg/dl)	TP (g/dl)
NC	110.7 ± 3.1^a^	106.9 ± 1.1^a^	75.2 ± 1.5^a^	14.2 ± 4.4^a^	7.3 ± 0.1^a^
DC	165.9 ± 6.3^d^	174.6 ± 1.2^d^	31.5 ± 1.6^d^	99.5±5^d^	3.5 ± 0.2^d^
SD	116.9 ± 3.6^ab^	122.5±3^b^	49.7 ± 1.6^b^	42.6 ± 1.5^b^	6.4 ± 0.1^bc^
PPS1	133.7 ± 2.1^c^	138.4 ± 1.1^c^	41.7 ± 1.2^c^	64.3 ± 3.4^ce^	6.1 ± 0.1^bc^
PPS2	127.2 ± 0.9^bc^	130.9 ± 1.7^c^	44.4 ± 1.5^bc^	56.6 ± 1.3^bc^	6.7 ± 0.2^b^
PM1	140.8 ± 2.1^c^	154.8 ± 0.9^e^	37.6 ± 1.3 cd	72.2 ± 2.8^e^	6 ± 0.1^c^
PM2	139.1 ± 0.8^c^	160.1 ± 1.8^e^	40.4 ± 1.3^c^	66.7 ± 1.7^ce^	6.4 ± 0.1^bc^

Note

^*^NC, Normal control; DC, Diabetic control; SD, Standard drug; PPS1, Peel–Seed (150 mg/kg); PPS2, Peel–Seed (200 mg/kg); PM1, Mucilage (150 mg/kg), PM2, Mucilage (200 mg/kg); TG, Triglyceride; HDL, High‐density lipoprotein; LDL, Low‐density lipoprotein; TP, Total protein. All data are expressed as mean ± SE. Means ± SE with different superscript (a, b, c, d, and e) in the same column differs significantly (*p* < .05).

## 4 DISCUSSIONS

The use of plant‐based natural medicine is a growing health issue owing to their wide range of nutritional and therapeutic value as a good source of vitamins, antioxidants, minerals, fibers, and bioactive metabolites. Since the primitive ages, various parts of plants, plant extracts, and plant‐based natural remedies have been utilized by all nations and civilizations to treat various ailments (Goni et al., 2021; Hossen et al., 2021; Islam et al., 2021; Khan et al., 2020). The present study followed the traditional method for the mucilage extraction procedure. Mucilage yield was quite low compared with other researches following different extraction procedure. In the current study, the yield of dry mucilage from okra was 0.5% on average where other studies showed 1.25%–4% dry mucilage yield from *Abelmoschus esculentus* (Chukwuma et al., 2018; Gemede et al., 2018). The variation in yield percentage may be due to the differences in extraction process, regional production processes, or weather condition of the production area. In terms of mineral content, both PM and PPS showed high amount of calcium, potassium, magnesium, sodium, and iron. Similarly, (Gemede et al., 2016) reported high amount of minerals in *Abelmoschus esculentus*. The herb used in this study is *Abelmoschus esculentus* mucilage, which is an ethnomedicinally important medicinal plant with a wide range of medicinal and nutritional properties. Aside from its nutritional content, many parts of the herb are widely utilized in traditional systems of medicine (antidiabetic, antipyretic, diuretic, antispasmodic, and so on) all over the globe but scientific basis of this herb is yet to be explored. The current study found that the mucilage of *Abelmoschus esculentus* has a strong and effective antidiabetic as well as antioxidant potential.

In the regulation of genes and the triggering of receptors, the physiological balance between free radicals and antioxidants is crucial. Moreover, an unfavorable alteration in free radical concentration can be damaging to the biological process, leading to diseases such as inflammation, neurological disorders, aging, and cancer. Antioxidants protect the body from the harmful effects of free radical damage and oxidative stress (Akter et al., 2021). It has been known that dietary flavonoids and antioxidants play vital role in antidiabetic mechanism in the body (Babu et al., 2013). As a result, researchers are paying more attention to natural compounds when it comes to discovering, screening, and characterizing antioxidant properties to replace synthetic ones. In terms of in vitro antioxidant activity, the phenol content, flavonoid content, and DPPH scavenging radical activity of both PM and PPS revealed their efficiency as an efficient antioxidant agent. In the present study, both PM and PPS extract significantly (*p* < .05) scavenged DPPH free radical. Previous studies have reported that okra seeds and peel both contain high amounts of polyphenolic compounds, including quercetin derivatives, catechins in seeds and quercetin, hydroxycinnamic acid derivatives in skins (Arapitsas, 2008). The present study also confirms the presence of phenolic and flavonoid content in terms of gallic acid equivalent and quercetin equivalent respectively in both mucilage and peel–seed mixtures. The presence of phenols and flavones demonstrates okra mucilage and peel–seed as a good source of antioxidants. A research study, (Gemede et al., 2018) also documented okra mucilage as a promising source of natural antioxidants. Active antioxidants in polysaccharides such as mucilage can reduce the blood glucose level in normal as well as drug‐induced diabetic subjects (Li et al., 2006). Studies have also shown that hydroxycinnamic acid, a derivative of cinnamic acid, can improve glucose hemostasis and insulin resistance, thus helping in the prevention of diabetes complications (Adisakwattana, 2017).

The present research demonstrated an elevated fasting blood glucose level in mice subjected to alloxan induction. By inhibiting the glucose sensor of beta cell known as glucokinase, alloxan impedes the secretion of glucose‐induced insulin. The considerable elevation in blood glucose levels was associated with a significant rise in TC, TG, LDL, and a reduction in HDL before *Abelmoschus esculentus* administration of alloxan‐induced diabetic mice. Blood glucose, TC, TG, and LDL of all examined mice were considerably reduced after *Abelmoschus esculentus* administrations, whereas HDL was increased. Alloxan induction in mice also exhibited typical visible feature of diabetes mellitus including weight loss, polydipsia (excessive thirst), and polyphagia (excessive hunger). Interestingly, reverse situation was observed after the treatment. Our findings show that mucilage and peel–seed of *Abelmoschus esculentus* have the capacity to be used as a natural oral medication with hypoglycemic and hypolipidemic effects.

The present experiment showed that raw mucilage and peel–seed mixture powder at both doses of 150 and 200 mg/kg significantly (*p* < .05) demonstrated the hypoglycemic effect of alloxan‐induced diabetic mice as well as ameliorated oral glucose tolerance and reduced the increased food and water intake. The magnitude of this reduction was found to be reliant on the dose of administration. The hypoglycemic effect of PM and PPS showed a proportionate relation with the increasing dose suggesting their usefulness in the treatment of diabetes mellitus. In terms of blood glucose level, after 3 weeks of PM and PPS administration, the dosage at 200 mg/kg differs marginally in degree when compared to standard drug glibenclamide administration at 5 mg/kg and is contrasted strongly with non‐treated diabetic control. A similar scenario was recorded in the case of glucose tolerance, where the blood glucose level in PPS2‐ and PM2‐treated mice after 120 min was found to be similar to the glibenclamide‐treated mice. Other treatments (PPS1 and PM1) also exhibited significantly lower blood glucose concentration after 2 h of glucose administration. The standard drug glibenclamide helps in diabetes management by controlling insulin secretion and insulin action (Luzi & Pozza, 1997). The underlying mechanism of PM and PPS in controlling blood glucose level may be similar to the mechanism of glibenclamide.

Hyperlipidemia is associated with coronary artery disease in diabetes patients (O'Brien et al., 1998). High blood glucose is correlated with a high risk of dyslipidemia. Hyperglycemia tends to increase triglyceride and LDL as well as decrease the HDL levels (Abbate & Brunzell, 1990). However, the present study exhibited the excess level of LDL, triglyceride, and total cholesterol in diabetic control group. Interestingly, the current study reveals that okra mucilage and peel–seed can help in the management of diabetes by controlling glycemic load in blood and thus helps in lowering the hyperlipidemic effect on diabetic mice. In addition, administration of PM and PPS showed (*p* < .05) a positive impact on lipid profile of diabetic mice. Moreover, total protein level in the treatment group has also significantly (*p* < .05) increased compared with diabetic group. These may be due to high protein content present in powdered mucilage and peel–seed which has also been shown in the current study. Hence, this study concludes that both PM and PPS have potential role in the treatment of dyslipidemia in diabetic subjects.

## 5 RESEARCH LIMITATION

The present research is limited to alloxan‐induced type 1 diabetes model and their glucose level and various biochemical parameters. Further research is required on various types of diabetic rodent model followed by the isolation and characterization of lead compounds along with their mechanism of actions. In addition, histopathological examination needs to be conducted to understand normal and distorted histological structure among different mice groups. In addition, extensive safety and toxicity study, pharmacodynamic and pharmacokinetic as well as inclusive cellular and molecular mechanistic research are suggested before going to clinical trials in the human model.

## 6 CONCLUSION

The findings of present study provide evidence that the mucilage of *Abelmoschus esculentus* possesses hypoglycemic and hypolipidemic effects in alloxan‐induced diabetic mice and promising antioxidant potential. Moreover, the results obtained from this study showed that *Abelmoschus esculentus* mucilage is rich in proteins and minerals. Mucilage also contains high amount calcium, potassium, magnesium, phosphorus, iron, and sodium. In conclusion, the *Abelmoschus esculentus* can be recommended as a potent source of hypoglycemic and hyperlipidemic drug candidates, as well as a potent natural antioxidant, in the treatment of diabetes. Furthermore, extensive research is strongly recommended on type 2 diabetic rodent model, followed by the isolation and identification of potent bioactive isolates.

## CONFLICT OF INTEREST

The authors declare that they do not have any conflict of interest.

## AUTHOR CONTRIBUTIONS

A.F.M Irfan Uddin Zim: Conceptualization (equal); Formal analysis (lead); Investigation (lead); Methodology (equal); Writing‐original draft (lead); Writing‐review & editing (equal). Jannatara Khatun: Conceptualization (equal); Funding acquisition (supporting); Methodology (equal); Project administration (lead); Supervision (lead); Writing‐original draft (supporting); Writing‐review & editing (equal). Mohammad Forhad Khan: Formal analysis (supporting); Investigation (equal); Methodology (supporting); Writing‐review & editing (equal). Md. Altaf Hossain: Conceptualization (supporting); Funding acquisition (supporting); Project administration (supporting); Writing‐review & editing (equal). Mohammad Mozibul Haque: Conceptualization (supporting); Formal analysis (supporting); Investigation (supporting); Methodology (equal); Project administration (supporting); Supervision (supporting); Writing‐original draft (supporting); Writing‐review & editing (equal).

## ETHICAL REVIEW

This study involves animal testing. This study was approved by the CVASU Institutional Animal Ethical Committee (Memo no.‐CVASU/Dir(R&E) EC/2019/39(2)). Whole studies were carried out in scrupulous guidelines for the care of laboratory animal.
